# Exploiting DNA repair defects in colorectal cancer

**DOI:** 10.1002/1878-0261.12467

**Published:** 2019-03-02

**Authors:** Nicole M. Reilly, Luca Novara, Federica Di Nicolantonio, Alberto Bardelli

**Affiliations:** ^1^ Fondazione Piemontese per la Ricerca sul Cancro ONLUS Candiolo Italy; ^2^ Candiolo Cancer Institute FPO‐IRCCS Candiolo Italy; ^3^ Department of Oncology University of Torino Candiolo Italy

**Keywords:** colorectal cancer, genome instability, homologous recombination, microsatellite instability, mismatch repair

## Abstract

Colorectal cancer (CRC) is the third leading cause of cancer‐related deaths worldwide. Therapies that take advantage of defects in DNA repair pathways have been explored in the context of breast, ovarian, and other tumor types, but not yet systematically in CRC. At present, only immune checkpoint blockade therapies have been FDA approved for use in mismatch repair‐deficient colorectal tumors. Here, we discuss how systematic identification of alterations in DNA repair genes could provide new therapeutic opportunities for CRCs. Analysis of The Cancer Genome Atlas Colon Adenocarcinoma (TCGA‐COAD) and Rectal Adenocarcinoma (TCGA‐READ) PanCancer Atlas datasets identified 141 (out of 528) cases with putative driver mutations in 29 genes associated with DNA damage response and repair, including the mismatch repair and homologous recombination pathways. Genetic defects in these pathways might confer repair‐deficient characteristics, such as genomic instability in the absence of homologous recombination, which can be exploited. For example, inhibitors of poly(ADP)‐ribose polymerase are effectively used to treat cancers that carry mutations in *BRCA1* and/or *BRCA2* and have shown promising results in CRC preclinical studies. HR deficiency can also occur in cells with no detectable BRCA1/BRCA2 mutations but exhibiting *BRCA*‐*like* phenotypes. DNA repair‐targeting therapies, such as ATR and CHK1 inhibitors (which are most effective against cancers carrying *ATM* mutations), can be used in combination with current genotoxic chemotherapies in CRCs to further improve therapy response. Finally, therapies that target alternative DNA repair mechanisms, such as thiopurines, also have the potential to confer increased sensitivity to current chemotherapy regimens, thus expanding the spectrum of therapy options and potentially improving clinical outcomes for CRC patients.

**Abbreviations**
ATRi: ataxia telangiectasia‐mutated and Rad3‐related inhibitors
BER: base excision repair
CHK1i: checkpoint kinase 1 inhibitors
CRC: colorectal cancer
DDR: DNA damage response
FA: Fanconi anemia
HR: homologous recombination
MMR: mismatch repair
MSI: microsatellite instability
MSS: microsatellite stable
PARPi: poly(ADP)ribose polymerase inhibitors

## Introduction

Colorectal cancer (CRC) is the third most common cancer worldwide and the second leading cause of cancer‐related deaths (Bray *et al*., 2018). In Europe, CRC accounts for the second highest number of cancer cases and deaths (Malvezzi *et al*., 2018), and in North America, CRC has the fourth highest rate of incidence and the second highest number of cancer‐related deaths (Jemal *et al*., 2017). While CRC death rates are slowly declining in the United States and Europe (Jemal *et al*., 2017; Malvezzi *et al*., 2018), the five‐year overall survival for patients with metastatic CRC (mCRC) remains poor (approximately 14.0%; NCI 2017). The standard chemotherapeutic regimen for mCRC is 5‐fluorouracil (5‐FU) in combination with either oxaliplatin (FOLFOX) or irinotecan (FOLFIRI) (Cremolini *et al*., 2015). These chemotherapy agents induce genotoxic damage in tumor cells that is recognized and repaired by DNA repair proteins (Helleday *et al*., 2008).

In 2012, The Cancer Genome Atlas (TCGA) conducted a comprehensive characterization of CRC tumors, including exome sequences, DNA copy numbers, and RNA expression levels (Network, 2012). Of the cases analyzed, 16% were classified as hypermutated (greater than 12 mutations per 10^6^ bases) and exhibited mutation enrichment in microsatellite regions indicating microsatellite instability (MSI) phenotype. The other 84% of cases were classified as microsatellite stable (MSS) and exhibited a higher frequency of somatic copy number alterations, suggesting chromosomal and subchromosomal defects (Network, 2012). The most frequently identified gene mutations in CRC tumors occur in *APC*,* TP53*, and *KRAS* (Huang *et al*., 2018; Wolff *et al*., 2018; Yaeger *et al*., 2018). Recent analysis of TCGA data identified mutations associated with DNA damage response genes and found that cases in the colon adenocarcinoma (COAD) and rectal adenocarcinoma (READ) datasets carried mutations in several DNA damage response and repair (DDR) genes (Knijnenburg *et al*., 2018).

Acquisition of mutations is a critical step for tumor development (Hanahan and Weinberg, 2011), and mutations that occur in DNA repair genes impair cells’ ability to restore damaged DNA and can lead to cell death or genome instability (Aguilera and Gomez‐Gonzalez, 2008). Mutations in MMR genes are observed in 2–3% of CRC patients (Lorans *et al*., 2018), while approximately 10% of CRC patients exhibit hypermethylation of *MLH1* (AlDubayan *et al*., 2018; Pearlman *et al*., 2017), contributing to a MMR‐deficient (MMRd) phenotype. The remaining CRC patient population can be classified as MMR‐proficient (MMRp). Defects in the MMR pathway are commonly used to classify CRCs, while mutations in HR and FA genes have been historically linked with breast and ovarian cancers (Hoang and Gilks, 2018, Knijnenburg *et al*., 2018). The TCGA‐COAD and TCGA‐READ PanCancer Atlas cohort (Cerami *et al*., 2012; Gao *et al*., 2013; Liu *et al*., 2018) includes mutational data for 528 patient tumor samples, and analysis of these samples identified 141 cases that carried mutations in at least one of 420 DNA repair genes. The majority of mutations identified were classified as ‘putative passenger’. While these mutations are not currently known to drive carcinogenesis, it is possible that the presence of these mutations will cause the cells to be DNA repair deficient. Using criteria that excluded likely passenger mutations, putative driver mutations were identified in 29 DNA damage response and repair genes (Table 1).

**Table 1:** Missense, truncating, and frameshift mutations (putative driver) in DNA damage response and repair genes identified in 528 colorectal cancer cases reported in The Cancer Genome Atlas Colon Adenocarcinoma (COAD) and Rectal Adenocarcinoma (READ) PanCancer Atlas datasets (Liu *et al*., 2018). Asterisk (*) indicates methylation data acquired from the TCGA‐COADREAD Provisional dataset

Gene	Cases affected (%)
TRP53	92 (17.4)
ATM	18 (3.4)
BRCA2	10 (1.9)
TP53BP1	9 (1.7)
MSH6	7 (1.3)
ATR	7 (1.3)
MTOR	5 (0.9)
SMARCB1	4 (0.8)
ATRX	3 (0.6)
BARD1	3 (0.6)
BLM	3 (0.6)
MSH3	3 (0.6)
BRIP1	2 (0.4)
FANCA	2 (0.4)
RAD50	2 (0.4)
EPC2	1 (0.2)
ERCC4	1 (0.2)
MLH1	1 (0.2)
	37 (10.3)*
MSH2	1 (0.2)
PMS2	1 (0.2)
RAD21	1 (0.2)
RAD21L1	1 (0.2)
RAD51C	1 (0.2)
SMC1A	1 (0.2)
XRCC2	1 (0.2)
XRCC3	1 (0.2)

Epigenetic modulation of gene expression can also lead to a repair‐defective phenotype. For example, hypermethylation of the *MLH1* promoter has been associated with the MSI phenotype in sporadic endometrial and hereditary nonpolyposis colorectal cancers (Esteller *et al*., 1998; Niv, 2007; Planck *et al*., 2003). Epigenetic down‐regulation of MMR genes has also been linked with resistance to alkylating chemotherapy agents in CRC tumor models (Planck *et al*., 2003), and studies have demonstrated that preventing down‐regulation of MMR genes can restore cellular sensitivity to these agents (Francia *et al*., 2005). Methylation data were not available for the PanCancer Atlas dataset; however, analysis of the TCGA‐COADREAD Provisional dataset (Cerami *et al*., 2012; Gao *et al*., 2013) determined that 10.3% of cases (37 out of 358) exhibited hypermethylation of *MLH1*. The presence of mutations or hypermethylation of promoter regions in one or more DNA repair genes in a CRC cell may contribute to a DNA repair‐defective phenotype that can be used to classify tumor subtypes and to choose an appropriate therapy regimen.

## DNA repair‐defective phenotypes in colorectal cancers

### Mismatch repair

**Figure 1:**
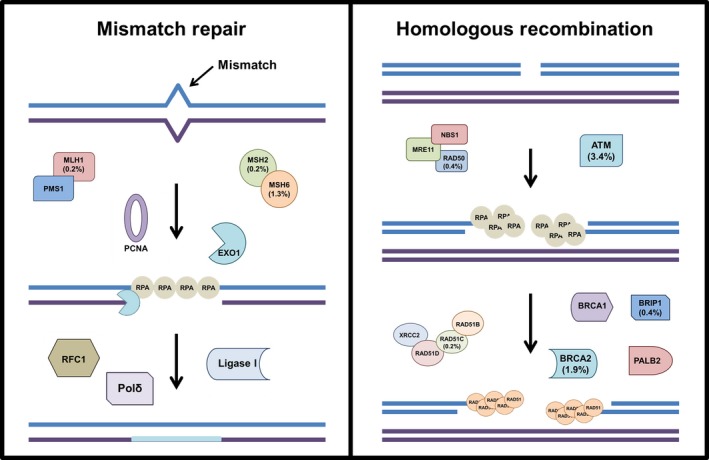
DNA repair pathways with mutated genes highlighted. Left panel: DNA mismatch repair pathway recognizes and removes incorrect DNA base pairs generated during replication. Right panel: homologous recombination proteins recognize and repair DNA double‐strand breaks. Key proteins in each pathway are shown and the percent of cases that carried mutations in these genes are listed (see Table 1 for details).

The MMR pathway recognizes and removes DNA base pair mismatches that occur due to replication errors (Iyer *et al*., 2006; Modrich, 2006) (Fig. 1; left panel). First, the mismatch is recognized by MutSα (MSH2/MSH6) and MutLβ (MLH1/PMS1) or MutSβ (MSH2/MSH3) and MutLα (MLH1/PMS2) heterodimer complexes that bind the DNA surrounding the mismatch. Downstream, the exonuclease EXO1 interacts with proliferating cell nuclear antigen (PCNA), initiating DNA resection in a 5′ to 3′ manner. Finally, polymerase δ replicates across the excised region and the DNA is ligated by DNA ligase I. Inactivating mutations in any of these genes decreases recognition of base pair mismatches, leading to increased mutational burden, particularly in microsatellite regions of the genome (Cortes‐Ciriano *et al*., 2017; Hause *et al*., 2016; Popat *et al*., 2005). One of the phenotypes exhibited in MMRd cells is MSI (Zeinalian *et al*., 2018), and several studies have shown that MSI corresponds with favorable prognosis and better survival (Popat *et al*., 2005; Thibodeau *et al*., 1993).

#### Microsatellite instability

A phenotype of cells that carry defects in mismatch repair is microsatellite instability (MSI), defined as high mutational burden in sequences along the genome that contain repetitive, short‐tandem sequences containing 1–6 nucleotide units up to 100 times, known as microsatellites (Zeinalian *et al*., 2018). The National Institutes of Health has defined five biomarkers that contain mono‐ or dinucleotide repeats in specific regions of the genome to be used for clinical determination of MSI status (Boland *et al*., 1998). PCR amplification of biomarker regions is performed, and the product size in tumor cells is compared with matched normal cells to determine whether mutations are present (Zeinalian *et al*., 2018). New advents in sequencing technology have allowed for the detection of MSI using whole‐exome sequencing (WES) data from tumor samples and normal tissue isolated from the same patient (Hause *et al*., 2016). A comprehensive analysis of microsatellite stability in 5930 exome samples demonstrated that these strategies are capable of distinguishing MSI cancer from MSS cancers, and these analyses identified genomic ‘hot spots’ that exhibit higher frequencies of MSI across cancer subtypes (Hause *et al*., 2016). Immunohistochemistry using antibodies against MLH1, MSH2, MSH6, and PMS2 is another effective technique that is used to assess MMR status (Yuan *et al*., 2015; Zeinalian *et al*., 2018).

Generation of neoantigens is another phenotype of MMRd cells that has been well characterized in CRC (Germano *et al*., 2017; Le *et al*., 2017; Scarpa *et al*., 2015). The defects in MMR machinery lead to frameshifts and indels that produce novel peptides within a cell. Once translated, these peptides can be exposed to the outside of the cell by the HLA proteins and activate immune surveillance of the tumor cells, thus making MMRd cells more susceptible to immune surveillance (Nakayama, 2014).

### Homologous recombination

The HR pathway is essential for preserving genome integrity in the event of DNA double‐strand breaks (DSBs) (Ranjha *et al*., 2018) (Fig. 1; right panel). If left unrepaired, this type of damage can lead to deletions, frameshifts, chromosome aberrations, and aneuploidy (Jackson and Bartek, 2009). During S phase, a DSB is first recognized by poly(ADP‐ribose) polymerase 1 (PARP1), a protein that scans the genome and detects DSB lesions (Ciccia and Elledge, 2010). PARP1 marks the damage site by attaching ADP‐ribose molecules to chromatin‐bound proteins surrounding the break (Haince *et al*., 2008). The ADP‐ribose units are essential for recruitment of meiotic recombination 11 (MRE11), RAD50, and Nijmegen breakage syndrome (NBS1) proteins, which form the MRN complex. The exonuclease activity of this complex produces single‐strand DNA (ssDNA) surrounding the break (Dodson *et al*., 2010; Haince *et al*., 2008; Huen *et al*., 2010; You and Bailis, 2010), and localization of MRN triggers ATM‐mediated signaling of downstream repair factors. Following MRN‐mediated resection, the single‐strand binding protein replication protein A (RPA) stabilizes the newly produced ssDNA overhangs (Marechal and Zou, 2015). Recruitment of BRCA1 and BRCA2, along with BRIP1, PALB2, and the RAD51B‐RAD51C‐RAD51D‐XRCC2 (BCDX2) complex, promotes RAD51 binding to the ssDNA overhangs (Candelli *et al*., 2014; Jensen *et al*., 2013; Short *et al*., 2016; Xu *et al*., 2017). Finally, RAD51 mediates strand invasion of the ssDNA overhang into a homologous DNA region, usually a sister chromatid, enabling repair to be completed (Qi *et al*., 2015).

#### Genome instability

In the context of distinct cancers types, such as breast and ovarian, ‘genome instability’ is typically attributed to defects in HR DNA repair genes (Burrell *et al*., 2013; Chien *et al*., 2015; Janssen *et al*., 2011; Vanderstichele *et al*., 2017). Hanahan and Weinberg classified ‘genome instability’ as an enabling characteristic of cancer and described how defects in DNA repair lead to loss of chromosomes, particularly at the telomere region (Hanahan and Weinberg, 2011). Genome instability is identified by structural alterations that include copy number variations and loss of heterozygosity, often observed in CRC cells (Druliner *et al*., 2018), and chromosomal rearrangements (Aguilera and Gomez‐Gonzalez, 2008). These elements are characteristic of HR defective cells, and specific genomic signatures have been identified in breast and ovarian cancer cells (Davies *et al*., 2017; Hillman *et al*., 2018; Vanderstichele *et al*., 2017). The detection of genomic rearrangements by whole‐genome sequencing in BRCA1/BRCA2‐deficient samples leads to identification of six distinct mutational signatures that correlated with BRCA status (Davies *et al*., 2017). Notably, the so‐called *BRCA‐ness* signature was also identified in cells that did not have detectable BRCA1/BRCA2 mutations, connecting genomic rearrangements with functional HR deficiency, and suggesting that additional molecular alterations might underline *BRCA*‐*like* phenotypes (Davies *et al*., 2017). *BRCA‐ness* mutational signatures in CRC tumors might be used as predictive biomarkers for HR deficiency regardless of BRCA status.

#### Telomere defects

In addition to genomic rearrangements, telomere length is a measurement of genome instability (Hackett *et al*., 2001). HR repair proteins function to protect telomere regions from damage (Claussin and Chang, 2015; Tarsounas *et al*., 2004), and telomere defects are often observed in genome unstable cells (Venkatesan *et al*., 2015). A recent study investigating telomere length in CRC determined that *KRAS*‐mutated cells exhibited extensive telomere shortening compared with control cells. In contrast, cells that carried *BRAF* mutations or were classified as MSI did not exhibit telomere defects (Balc'h *et al*., 2017). Another independent study analyzed telomere length in precursor colorectal lesions and observed that telomere shortening was associated with mitochondrial microsatellite instability in the tumor tissue samples and with *KRAS* and *BRAF* mutations in the normal tissues (Park *et al*., 2017). Studies have also demonstrated that *KRAS‐*mutated CRC cells can become dependent on RAD51‐mediated repair (Kalimutho *et al*., 2017), a key protein in the HR pathway that is essential for maintaining telomere integrity (Badie *et al*., 2010; Le *et al*., 1999; Lu *et al*., 2014; Signon *et al*., 2001). Together, these data suggest that telomere shortening is indicative of DNA repair defects and may be a biomarker of early CRC carcinogenesis.

## Targeting DNA repair in colorectal cancer

**Figure 2:**
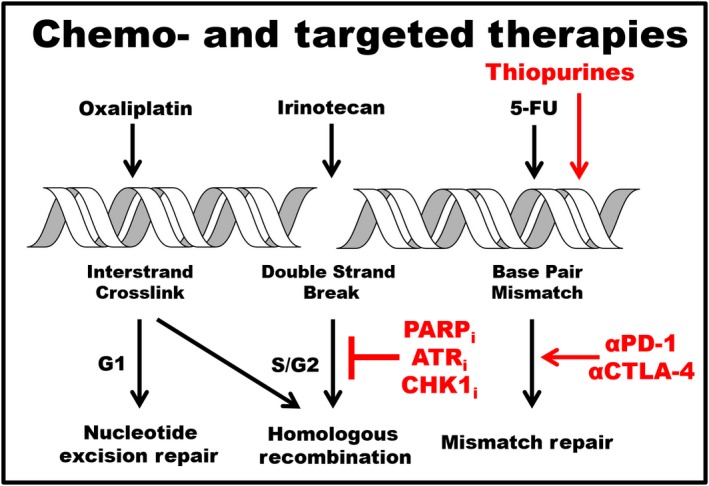
Therapies targeting cancer specific DNA repair defects. Current chemotherapy agents (black) used to treat mCRC. Oxaliplatin induces DNA interstrand cross‐links that are repaired by nucleotide excision repair proteins during G1 and by Fanconi anemia and HR proteins during S phase. Irinotecan (SN38) is a topoisomerase inhibitor that induces single (SSB)‐ and double‐strand breaks (DSBs) that are repaired by HR and BER proteins. 5‐Fluorouracil is an antimetabolite that can lead to DNA base pair mismatches repaired by the MMR pathway. Alternative therapies (red) that can be used in combination with current chemotherapy agents. PARPi and ATRi induce stalled replication forks and DSBs that are lethal in cells carrying mutations in HR genes, and CHKi block cell cycle arrest in the presence of replication stress. Chemosensitivity can be further induced in cells treated with genotoxic agents in combination with targeted therapies. Thiopurines induce DNA base pair mismatches that can lead to increased mutational and neoantigen burdens. Anti‐PD‐1 and CTLA‐4 immunotherapies target MMRd CRC tumors and are most effective against tumors with high neoantigen burdens.

Current medical regimens for CRC patients include combination therapies with oxaliplatin, irinotecan, and 5‐FU (Cremolini *et al*., 2015). These ‘genotoxic’ drugs directly or indirectly induce DNA damage that is recognized by specific repair pathways (Fig. 2). Oxaliplatin is a platinum‐based compound that can induce cell death through several mechanisms, such as inducing ribosome biogenesis stress (Bruno *et al*., 2017). The genotoxic activity of this drug is attributed to its ability to bind the N7 of guanine nucleotides in DNA, generating interstrand cross‐links that can inhibit replication during S phase (Ray *et al*., 2018). Irinotecan, a camptothecin analog, binds to topoisomerase I and DNA, preventing dissociation of topoisomerase I during S phase and ultimately leading to DNA DSBs (Li *et al*., 2017). These two types of DNA damage are recognized and repaired by the FA/HR pathways (Ceccaldi *et al*., 2016). 5‐FU is an antimetabolite that inhibits thymidylate synthase, an enzyme involved in nucleotide synthesis, and is thought to inhibit DNA replication thus leading to abasic sites that are repaired by base excision repair (BER) proteins (Huehls *et al*., 2016). 5‐FU can also be incorporated into DNA, resulting in DNA mismatches that are recognized and repaired by the MMR pathway (Iwaizumi *et al*., 2011). An alternative nucleotide analog, TAS‐102, has been approved by the FDA as a treatment option for mCRC patients (Marcus *et al*., 2017). TAS‐102 inhibits nucleoside synthesis in a similar mechanism of action to the standard therapy 5‐FU and is effective in treating patients that are refractory to 5‐FU therapy (Lenz *et al*., 2015). Recent studies have linked mutations in DNA repair genes, specifically those associated with HR, as predictive markers of the efficacy of TAS‐102 in patients (Suenaga *et al*., 2017).

These compounds are most effective in cells with defects in the respective repair pathways, and CRC patients that carry mutations in repair‐associated genes can be predicted to respond well to these types of therapies.

### Immune checkpoint blockade

Colorectal cancer tumors that have increased neoantigen production due to MMR deficiency also have higher levels of tumor infiltrating lymphocytes (TILs) and increased programmed death ligand‐1 (PDL‐1) protein expression (Germano *et al*., 2018; Kim *et al*., 2016; Llosa *et al*., 2015). Recent studies have shown that MSI tumor cells are responsive to PD‐1 and CTLA‐4 immune blockade (Germano *et al*., 2017; Le *et al*., 2017; Luksza *et al*., 2017; McGranahan *et al*., 2016). In one study, patients with higher TILs and PD‐L1 expression responded to checkpoint blockade better compared with patients with lower PD‐L1 expression, suggesting that this phenotype can be used as a predictor of therapy response (Wang *et al*., 2018). A 2017 Phase II clinical trial of nivolumab, a PD‐L1 immune checkpoint inhibitor, in patients with MMRd and MSI‐H CRC who had received at least three rounds of prior therapy and were no longer responsive to first‐line treatments found that this therapy provided durable response and disease control in these patients. Of the 74 patients enrolled, 31% achieved objective response and 51% achieved disease control (Overman *et al*., 2018). Based on these data, nivolumab was given FDA approval for the treatment of mCRC with MSI‐H or MMRd tumors (Sarshekeh *et al*., 2018). In addition to CRC, studies have demonstrated that solid tumors of multiple tissue types exhibiting MSI show durable response to PD‐L1 blockade, and based on this evidence, the FDA approved these agents for use in any cancers that histologically exhibit MSI (Lemery *et al*., 2017).

The MMRd tumors have increased mutational burden (TMB), a phenotype that can also be observed in a small subset of tumors that are MSS. One study analyzed over 6000 CRC cases, 95% of which were classified as MSS, and observed that 2.9% of MSS tumors exhibited high TMB and, within this subset of tumors, 54% responded to anti‐PD‐L1 immunotherapy (Fabrizio *et al*., 2018). The results of this study suggest that in MMRd tumors that do not exhibit MSI, TMB can be used as a predictor for therapy response to tumor checkpoint inhibition.

### PARP inhibitors

In the presence of directly induced DNA breaks, PARP1 poly(ADP‐ribosyl)ates chromatin surrounding the damage to initiate activity of downstream HR proteins (Haince *et al*., 2008). Additionally, PARP functions to regulate replication fork progression and maintain fork stability. Chemical inhibition of PARP activity can interfere with either of these activities, leading to replication fork collapse or accelerated fork progression that generates DNA single‐ and double‐strand breaks (D'Andrea, 2018; Maya‐Mendoza *et al*., 2018). Poly‐(ADP) ribose polymerase inhibitors (PARPi) have been used as anticancer agents since the early 2000s (McCabe *et al*., 2006), and the first PARPi, olaparib, was approved for *BRCA*‐mutated ovarian cancer in 2014 (Kim *et al*., 2015). Approval of these drugs was first given for the treatment of breast and ovarian cancers, and studies found that this therapy regimen was most effective in cells that carry functional defects in DNA DSB pathways, most notably in *BRCA‐*mutated cells (Cortesi *et al*., 2018; Ghiringhelli *et al*., 2016; Lin *et al*., 2014; Lord and Ashworth, 2017; Mittica *et al*., 2018; Sunada *et al*., 2018). Recent studies ascribed this synthetic lethality phenotype to the loss of PARP activity at replication forks, suggesting that PARP inhibition promotes rapid fork progression, leading to increased genome instability that the cell cannot overcome when HR defects are also present (Maya‐Mendoza *et al*., 2018).

Early investigations into the use of PARPi for the treatment of CRC began with the inhibitor ABT‐888, later known as veliparib. Prior studies had demonstrated that ABT‐888 was effective in *BRCA*‐deficient cells compared with proficient counterparts and that response to PARPi was further increased when combined with platinum‐based genotoxic compounds (Clark *et al*., 2012). Following on the premise that PARPi will increase sensitivity to genotoxic compounds in cancer cells, the effect of ABT‐888 in combination with irinotecan in CRC cells was investigated (Davidson *et al*., 2013). This study observed a synergistic response to irinotecan or oxaliplatin in combination with ABT‐888 in CRC cells (Davidson *et al*., 2013). Another study demonstrated that addition of ABT‐888 increased sensitivity of CRC cells to radiation (Shelton *et al*., 2013), further supporting the hypothesis that PARPi are a viable option to improve response to current therapy regimens in CRC. A recent phase II open‐label study evaluated the efficacy of the veliparib PARPi in combination with temozolomide in mCRC patients that were refractory to standard therapies. Fifty patients were enrolled in the trial, and 24% exhibited disease control response and 4% showed partial response to the combination therapy (Pishvaian *et al*., 2018).

Synthetic lethality has been clearly demonstrated in cells that harbor defects in DNA DSB repair pathways, specifically *BRCA‐*mutated cells (Lord and Ashworth, 2017; McCabe *et al*., 2006). For this reason, it can be predicted that CRC cells that respond well to PARPi most likely carry defects in DSB repair proteins. One study found that CRC cells carrying inactivating mutations in *ATM* have increased sensitivity to the PARPi olaparib (Wang *et al*., 2017). These data correlate well with earlier studies in gastric and lung cancers that found that loss of ATM protein expression increased cellular sensitivity to PARP inhibition (Kubota *et al*., 2014; Schmitt *et al*., 2017). However, due to the limited number of preclinical models studied, these results should be confirmed in larger cohorts.

In addition to exploiting DSB repair defects in CRC cells, it has been postulated that cells that exhibit MSI may also be susceptible to PARP inhibition. In 2014, a study demonstrated that loss of MRE11 in MSI CRC cells increased cellular sensitivity to ABT‐888 (Vilar *et al*., 2011). One proposed explanation is the MSI induces mutations within DNA repair genes, conferring a repair‐deficient phenotype and making the cells more susceptible to the effects of PARP inhibition. This hypothesis has been supported in models of myeloid malignancies (Gaymes *et al*., 2013). More recently, a phase II clinical trial investigated off‐label of use of PARPi in MSS and MSI CRCs to determine whether microsatellite status was a predictive marker of PARPi response. The results of this study suggested that PARPi alone did not affect patient outcomes regardless of microsatellite status (Leichman *et al*., 2016). While PARPi have been approved for the treatment of other cancer types that exhibit HR deficiency, PARPi are not currently used for CRC patients.

As discussed above, HR deficiency can also occur in cells with no detectable BRCA1/BRCA2 mutations but showing *BRCA*‐*like* phenotypes. Accordingly, it will be of interest to assess whether the *BRCA‐ness* mutational signatures might be used as predictive biomarkers for sensitivity to PARP inhibitors and oxaliplatin.

#### DNA repair‐mediated resistance mechanisms to PARP inhibition

The DNA repair‐associated resistance mechanisms to PARP inhibition have been well characterized in breast and ovarian cancers (D'Andrea, 2018), and it is reasonable to predict that similar mechanisms may promote resistance in CRC patients following PARP blockade. One mechanism is re‐activation of HR activity, either through acquired mutations in DNA repair genes or through increased activity of effector proteins that promote HR activity. Acquired mutations have also been described in HR genes that restore the reading frame and expression of the protein following exposure to PARPi (Quigley *et al*., 2017). Restoration of BRCA1 expression reverses HR‐mediated repair deficiency and allows the cells to repair the damage induced through PARP inhibition (D'Andrea, 2018), and mutations that restore activity of other HR proteins have also been observed in PARPi‐resistant cancer cells. A study of 12 pretreatment and postprogression patient samples observed the acquisition of mutations in the *RAD51D* and *RAD51C* genes that restored protein expression and promoted resistance to rucaparib (Kondrashova *et al*., 2017), and an independent study identified a point mutation in the *XRCC2* DNA repair gene that decreased sensitivity of CRC cells to olaparib (Xu *et al*., 2014). In addition to mutations that restore gene expression, epigenetic regulation of gene expression can predict response and resistance to PARPi. In a study of 12 high‐grade serous ovarian cancer (HGSOC) patient‐derived xenografts and 21 patient samples (ARIEL2 trial), response and resistance to rucaparib were correlated with methylation status of *BRCA1*. Methylation‐mediated silencing of all *BRCA1* copies predicted response to rucaparib, while heterozygous methylation was associated with resistance to therapy (Kondrashova *et al*., 2018).

Homologous recombination activity can also be increased through regulatory effector proteins, such as the demethylase JMJD1C. These enzymes target MDC1 for demethylation at Lys45 to promote its interaction with RNF8 and its function in the HR signaling cascade (Watanabe *et al*., 2013). Overexpression of JMJD1C has been detected in colon cancer tissues compared with normal tissues (Chen *et al*., 2018). Furthermore, depletion of JMJD1C in cells induces cellular resistance to ionizing radiation (IR) and PARPi (Watanabe *et al*., 2013). These data suggest that resistance mechanisms can arise from regulatory proteins in DNA repair pathways and further show that a comprehensive understanding of repair efficiency is necessary to properly predict therapy response.

A second mechanism of PARPi resistance described in *BRCA*‐mutated cancers is increased activity of alternative DNA repair pathways. In the absence of BRCA1, activity of nonhomologous end joining (NHEJ), an alternative DNA DSB repair pathway, is increased specifically through loss of p53 binding protein 1 (53BP1) expression (Bouwman *et al*., 2010; Jaspers *et al*., 2013). 53BP1 regulates pathway choice in response to DNA DSBs and promotes NHEJ activity through inhibition of BRCA1 recruitment during early DSB repair (Bakr *et al*., 2016). Somatic loss of 53BP1 expression in *BRCA1*‐mutated cancers leads to partial restoration of HR‐mediated DSB repair and contributed to resistance to PARPi (Jaspers *et al*., 2013). A recent study observed that cancer cells carrying mutations that lead to expression of a truncated BRCA1 protein, which maintained the ability to interact with PALB2, still developed PARPi resistance even in the absence of 53BP1. In contrast, loss of the interaction between BRCA1 and PALB2 did not confer PARPi resistance when 53BP1 expression was decreased, suggesting that this protein interaction is required for any measurable HR activity (Nacson *et al*., 2018).

Increased activity of NHEJ in the absence of BRCA1 can also be attributed to expression of REV7, the noncatalytic subunit of DNA polymerase ζ, which functions to promote translesion synthesis in the presence of DNA damage (Lee *et al*., 2014). In response to DSBs, REV7 interacts with 53BP1 to prevent DNA‐end resection at the break site. This activity promotes end‐ligation mediated by NHEJ proteins and contributes to DSB repair following PARP inhibition (Xu *et al*., 2015). REV7 also functions as part of the Shieldin complex that promotes 53BP1 mediated NHEJ in *Brca1*‐deficient cells (Ghezraoui *et al*., 2018), a function that could potentially contribute to PARP inhibitor resistance. These activities in the presence of DSBs induced through PARP inhibition provide mechanisms to overcome the damage, leading to resistance to PARP targeting therapies.

A third mechanism of resistance to PARPi, specifically in HR‐deficient cancer cells, is restoration of replication fork stability. One study demonstrated that reduced recruitment of the exonuclease MRE11 in BRCA1‐deficient cells prevented end resection at these sites and promoted fork stability (Ray Chaudhuri *et al*., 2016). Reduced recruitment of another DNA exonuclease, MUS81, has also been shown to promote stability of replication forks in BRCA2‐deficient cancers that develop resistance to PARP inhibition (Rondinelli *et al*., 2017). Finally, it has been reported that maintenance of replication forks can be regulated at the transcriptional level. One study observed that BRCA2‐deficient cells treated with PARPi overcome therapeutic pressure by down‐regulating expression of the transcription repressor E2F7. One of the genes under the control of E2F7 is RAD51, and loss of E2F7 expression increases expression of RAD51, enhancing HR activity even in the absence of BRCA2 (Clements *et al*., 2018).

Resistance to PARP inhibition can also arise from altered activity of PARP and PARP‐associated proteins. One example is loss of poly(ADP‐ribose) glycohydrolase (PARG) that has been shown to be a major resistance mechanism to PARP inhibition in *Brca2*‐mutated cells (Gogola *et al*., 2018). Under unperturbed cellular conditions, PARG functions to remove poly(ADP‐ribose) chains generated by PARP1 at the site of DNA damage. PARP inhibitors can either trap PARP1 on the chromatin, leading to stalled replication forks and DSBs, or can inhibit the enzymatic activity and prevent generation of poly(ADP‐ribose) polymers that signal HR‐mediated DSB repair (Dziadkowiec *et al*., 2016). In the case of the former, PARPi are most effective when PARG is still active in order to remove the poly(ADP‐ribose) polymers and inhibit HR signaling. When PARG activity is lost, the polymers are still present and HR‐mediated repair is still functional. This residual HR activity can counteract the effect of the PARPi and lead to resistance (Gogola *et al*., 2018).

Together, these studies describe mechanisms of resistance associated with re‐activation of HR activity and provide evidence describing how activity of multiple repair pathways can contribute to resistance to DNA repair targeted therapies.

## Alternative DNA repair‐targeting therapies for use in colorectal cancer

The most common chemotherapy agents used for the treatment of CRC induce DNA damage that is recognized and repaired by DNA repair pathways. Inherent defects in these pathways make CRC cells more sensitive to these treatments, and for tumors that do not harbor mutations in DNA repair‐associated genes, these pathways can be targeted to induce a repair‐defective phenotype. The therapies described in the previous section either target or exploit characteristics of DNA repair‐deficient CRC cells. However, there are still other alternative therapies that target DNA repair mechanisms that have not been studied in the context of CRC (Gavande *et al*., 2016). In this section, we will describe alternative, and potentially novel, treatment regimens that can either take advantage of repair defects in CRC cells or induce repair deficiency in CRC tumors and thus make those cells more responsive to current chemotherapy agents.

### ATR inhibitors

Colorectal adenomas exhibit endogenous replication stress (Bartkova *et al*., 2005), a phenotype that can be exploited through therapies that target replication stress signaling proteins (Halazonetis *et al*., 2008). One target under investigation is the ataxia telangiectasia and Rad3‐related (ATR) protein. ATR functions at the sites of replication forks and is essential for signaling repair proteins when a cell experiences stress due to DNA damage that blocks replication progression. ATR directly interacts with RPA that coats single‐strand DNA generated during replication, and this ability allows ATR to sense stalled replication forks and corresponding DNA damage (Zou and Elledge, 2003). Additionally, ATR functions in conjunction with ATM in response to IR and is required for promoting accurate repair of DNA DSB damage (Marechal and Zou, 2013). Defects in replication fork protection are correlated with sensitivity to ATR inhibitors (ATRi), and patients who do not exhibit defects in HR but have unstable replication forks may benefit from ATRi therapies (Hill *et al*., 2018). Furthermore, ATRi is also a viable option to target BRCA‐deficient cancer cells that have acquired resistance to PARPi, by inhibiting the ‘rewired’ HR pathway that is promotes resistance to PARP inhibition (Haynes *et al*., 2018; Yazinski *et al*., 2017). For these reasons, ATR is an attractive target to disrupt DNA repair in cancer cells.

Early investigations into ATRi were performed in breast and ovarian cancer cells. Several studies have identified a synthetic lethality with ATRi and ATM or p53 deficiency (Reaper *et al*., 2011; Toledo *et al*., 2011), and this effect is further increased when cells are also treated with genotoxic agents (Reaper *et al*., 2011; Shi *et al*., 2018). One study reported that the ATRi NU6027 sensitized cells to cisplatin in wild‐type p53 and functional MMR expressing cells, while mutant p53 cells with functional MMR were most sensitive to temozolomide in combination with NU6027 (Peasland *et al*., 2011). In addition, ATR inhibition was synthetic lethal in combination with PARPi or in cells that had defective HR (through loss of XRCC1) (Peasland *et al*., 2011; Sultana *et al*., 2013). Interestingly, one study reported that inhibition of ATR in BRCA1‐depleted cells further sensitized the cells to damage induced by cisplatin and veliparib, suggesting that ATR inhibition functions independently of BRCA status (Huntoon *et al*., 2013). More recent studies have shown that pancreatic ductal adenocarcinoma (PDAC) and various gastrointestinal cancer cells that exhibit loss of ATM were more sensitive to ATRi (Min *et al*., 2017; Perkhofer *et al*., 2017). One study demonstrated that the ATR inhibitor AZD6738 induces a synthetic lethal phenotype in ATM‐deficient, but not ATM‐proficient, gastric cancer cells, and *in vivo* tumor growth of ATM‐deficient gastric cell xenografts was effectively controlled by treatment with AZD6738 compared with control (Min *et al*., 2017).

Recently, DNA DSB repair has been implicated in regulating the expression of PD‐L1 in cancer cells (Sato *et al*., 2017), and the effect of ATR inhibition on PD‐L1 expression, and consequently on immune surveillance of tumor cells, has been investigated (Sun *et al*., 2018; Vendetti *et al*., 2018). A siRNA‐mediated screen of DSB repair genes found that loss of BRCA2 enhanced expression of PD‐L1, specifically in response to DSBs induced by IR or PARPi. Furthermore, loss of genes associated with the error‐prone NHEJ pathway, such as Ku80, substantially enhanced PD‐L1 expression in response to IR (Sato *et al*., 2017). It was observed that treatment with IR and cisplatin significantly increased expression of PD‐L1 and that this effect was abrogated when cells were also treated with pharmacological inhibitors of ATR. Additionally, decreased PD‐L1 expression in the presence of ATRi led to increased immune surveillance of tumor cells, and controlled tumor growth. These data suggest that ATRi would be effective in MMRp cells that have increased PD‐L1 expression as a mechanism of overcoming immune evasion and re‐activating the immunogenicity of these tumor cells (Sun *et al*., 2018).

Together, these data suggest that ATR inhibition is most effective when combined with genotoxic agents and support the hypothesis that DNA repair‐defective CRC cells may also experience synthetic lethality when treated with ATRi. It would be interesting to test ATRi in MMRd preclinical models of mCRCs that are able to evade immune surveillance despite high levels of neoantigens.

### CHK1 Inhibitors

Another key player in the DNA damage response signaling cascade is checkpoint kinase 1 (CHK1) that directly interacts with ATR in the presence of replication stress during S phase and promotes replication fork stabilization (Chen and Poon, 2008). Following the same principle as ATRi, inhibitors targeting CHK1 (CHK1i) have been developed to inhibit replication stress signaling in cancer cells that already exhibit DNA repair defects.

Prexasertib is one CHK1i that has been thoroughly investigated in patients with squamous cell carcinomas (Hong *et al*., 2018), non‐small‐cell lung carcinomas (Sen *et al*., 2017), and high‐grade serous ovarian carcinomas (Brill *et al*., 2017; Lee *et al*., 2018), often in combination with other therapies. Early studies demonstrated that prexasertib (LY2606368) induced replication catastrophe and DNA damage while concomitantly disabling cell cycle checkpoints, ultimately leading to apoptosis. This effect was observed *in vitro* and *in vivo* in models of acute lymphoblastic leukemia and squamous cell carcinoma (Ghelli Luserna Di Rora *et al*., 2016; King *et al*., 2015).

In the context of CRC, one study investigated the effect of CHK1 inhibition on CRC stem cells (Manic *et al*., 2018). The authors observed that treatment with prexasertib, both *in vitro* and *in vivo*, inhibited replication and disabled cell cycle checkpoints, causing the cells to enter mitosis prematurely, ultimately leading to apoptosis. Interestingly, this effect was observed in cells that harbored *KRAS* mutations, a subset of CRCs particularly difficult to target and treat (Manic *et al*., 2018). An independent study also observed efficacy of CHK1 inhibition in *KRAS‐*mutated lung and colon adenocarcinoma cells, especially when combined with MK2 inhibitors (Dietlein *et al*., 2015). MK2 functions in a pathway parallel to CHK1 and is responsible for maintaining cell cycle checkpoints in response to stress (Reinhardt *et al*., 2010). *KRAS*‐mutated cells exhibit intrinsic genotoxic stress that leads to constant activation of CHK1 and MK2, and inhibition of these proteins induced mitotic catastrophe *in vitro*, in murine cancer models, and in patient‐derived cells (Dietlein *et al*., 2015). CHK1 inhibitors might be tested in *KRAS*‐mutated CRC, a subset that currently has limited therapy options.

### Thiopurines

Thiopurines are a class of nucleotide analogs that have been used successfully for the treatment of childhood leukemias (Karran, 2006). Thiopurines are incorporated into DNA during replication, leading to DNA base pair mismatches that are removed by MMR proteins (Coulthard and Hogarth, 2005; Karran, 2006; Munshi *et al*., 2014). The effect of the thiopurine analog 6‐thioguanine (6TG) on cell growth has been tested in CRC cell models. MMRd CRC cell lines are resistant to 6TG compared with MMRp counterparts (Carethers *et al*., 1996; Yan *et al*., 2003). 6TG induces reactive oxygen species (Brem and Karran, 2012) can trigger activity of BER proteins, suggesting that BER‐deficient cells may have increased cellular sensitivity to thiopurines. Further studies have also demonstrated that HR and FA proteins function to repair thiopurine induced damage (Brem and Karran, 2012) and that cells deficient for HR proteins, such as RAD51D, have increased sensitivity to thiopurines (Rajesh *et al*., 2011). These data suggest that this class of compounds might be effective in CRC tumors that are MMRp but carry mutations in genes associated with HR and BER.

## Clinical trials of DNA repair inhibitors in colorectal cancer

Targeting DNA repair in CRC has the potential to further increase the efficacy of current therapies, and, as described above, there have been multiple preclinical studies investigating DNA repair‐targeting therapies in CRC. None of these therapies have been approved by the FDA for use in CRC patients; however, several trials are ongoing (Table 2). To date, six clinical trials investigating the efficacy of PARPi in CRC are ongoing or have been completed. Of the five completed trials, only NCT00912743 has reported results (Leichman *et al*., 2016). In this study, the efficacy of olaparib was investigated in 33 CRC patients stratified by microsatellite status. Thirteen MSI‐H and 20 non‐MSI‐H patients were enrolled and treated with olaparib 400 mg twice a day. The median of progression‐free survival (PFS) was 61 days for the MSI‐H cohort and 55 for the non‐MSI‐H cohort. Overall survival (OS) was reported to be 248 days for the MSI‐H cohort and 209.5 days for the non‐MSI‐H. There was no statistical significance in the median PFS or OS for non‐MSI‐H cohort. The results of this study suggest that olaparib alone is ineffective in CRC patients regardless of microsatellite status, and the authors recommend that further studies investigate the use of olaparib in combination with DNA damaging agents for this patient cohort (Leichman *et al*., 2016). Importantly, this study was conducted in CRC patients that were not enriched for HR deficiency status.

**Table 2:** Clinical trials of PARP, ATR, and CHK1 inhibitors reported to the U.S. National Library of Medicine. (https://clinicaltrials.gov/ct2/home)

Trial identifier	Therapy	Disease(s)	Status
Clinical trials in colorectal cancer			
NCT00912743	Olaparib	Chemorefractory metastatic colorectal cancer	Completed Results Available (Leichman *et al*., 2016)
NCT02484404	Olaparib Cediranib MEDI4736	Ovarian, triple negative breast, lung, prostate, colorectal cancers	Recruiting
NCT02305758	FOLFIRI Bevacizumab Veliparib	Untreated metastatic colorectal cancer	Completed (Gorbunova *et al*., 2018)
NCT01051596	Temozolomide ABT‐888	Colorectal cancer	Completed (Pishvaian *et al*., 2018)
NCT01589419	Veliparib Capecitabine Radiation	Locally advanced rectal cancer	Completed (Czito *et al*., 2017)
NCT02033551	Veliparib Carboplatin Paclitaxel FOLFIRI	Metastatic and chemorefractory Breast cancer, ovarian cancer, colon cancer, lung cancer, gastric cancer, solid tumors	Completed (Berlin *et al*., 2018)
Clinical trials in other cancers			
NCT03669601	AZD6738 Gemcitabine	Cancer	Not yet recruiting
NCT03682289	AZD6738 Olaparib	Clear cell renal cell carcinoma, Pancreatic ductal adenocarcinoma, Renal cell carcinoma cancers	Not yet recruiting
NCT03428607	AZD6738 Olaparib	SCLC	Not yet recruiting
NCT02630199	AZD6738 Paclitaxel	Refractory cancers	Recruiting
NCT03462342	AZD6738 Olaparib	High‐grade serous carcinoma	Recruiting
NCT02223923	AZD6738	Solid tumor refractory to conventional therapy	Suspended
NCT01955668	AZD6738	Chronic lymphocytic leukemia, prolymphocytic leukemia, B‐cell leukemia	Completed
NCT02264678	AZD6738 Carboplatin Olaparib MEDI4736	Advanced solid malignancies—H&N SCC, ATM Pro/Def NSCLC, gastric and breast cancer	Recruiting
NCT03328273	AZD6738 Acalabrutinib	Chronic lymphocytic leukemia	Recruiting
NCT03330847	AZD6738 Olaparib	Metastatic triple negative breast cancer	Recruiting
NCT03022409	AZD6738 Olaparib	Head and neck squamous cell carcinoma	Recruiting
NCT02576444	AZD6738	Cancer	Recruiting
NCT03527147	AZD6738	NHL, DLBCL, non‐Hodgkin's lymphoma, diffuse large B‐cell lymphoma	Recruiting
NCT03334617	AZD6738	Non‐small‐cell lung cancer	Recruiting
NCT02937818	AZD6738 Olaparib	Platinum refractory extensive‐stage small cell lung carcinoma	Recruiting
NCT02203513	LY2606368	Breast, ovarian, prostate	Recruiting (Lee *et al*., 2018)
NCT01870596	SCH900776	Acute myeloid leukemia	Completed
NCT03495323	LY3300054 Prexasertib	Cancer	Recruiting
NCT02808650	Prexasertib	Childhood solid neoplasm, Recurrent central nervous system neoplasm, recurrent malignant solid neoplasm, refractory central nervous system neoplasm, refractory malignant solid neoplasm	Recruiting
NCT02797964	SRA737	Advanced solid tumors or non‐Hodgkin's lymphoma	Recruiting
NCT02797977	SRA737 Gemcitabine Cisplatin	Advanced solid tumors	Recruiting
NCT02873975	LY2606368	Advanced cancers	Recruiting
NCT03057145	LY2606368 Olaparib	Solid tumor	Recruiting
NCT01115790	Prexasertib	Advanced cancer, squamous cell carcinoma, carcinoma, squamous cell of head and neck, lung squamous cell carcinoma, anal squamous cell carcinoma, carcinoma, non‐small‐cell lung	Completed
NCT01139775	LY2603618 Pemetrexed Cisplatin	Non‐small‐cell lung cancer	Completed
NCT02735980	Prexasertib	Small cell lung cancer	Completed
NCT02514603	Prexasertib	Neoplasm	Completed
NCT03735446	Prexasertib Mitoxantrone Etoposide Cytarabine	Acute myeloid leukemia, Myelodysplastic syndromes	Not yet recruiting
NCT03377556	Talazoparib	Squamous cell lung cancer	Recruiting

Multiple clinical trials investigating the efficacy of the ATRi AZD6738 have been initiated, and most of these include gastrointestinal malignancies other than CRC. NCT01955668 is the only trial that has been completed thus far, and the results of this study have not been reported in full. Currently, the majority of clinical trials for AZD6738 focus on assessing the efficacy and safety of the drug in patients. As yet, there have not been any trials initiated to further elucidate sensitivity of specific cancer subtypes, such as *ATM*‐deficient tumors, in the clinical setting. In addition to analyzing the efficacy of AZD6738 alone, several studies are investigating the efficacy of this drug in combination with the PARPi olaparib, particularly in patients who were refractory to primary therapies. Once completed, these studies have the potential to describe novel therapy regimens can be used to treat CRC patients.

Multiple clinical trials with CHK1 inhibitors are ongoing. One trial (NCT02203513) has reported results from a cohort of *BRCA* wild‐type high‐grade serous ovarian cancer patients treated with the CHK1 inhibitor prexasertib (Lee *et al*., 2018). In this cohort, 80% of the patients were platinum‐resistant or refractory at the start of the trial. Sixteen patients (out of 28) exhibited partial response during the treatment time and one patient died during the study due to tumor progression (Lee *et al*., 2018). Preclinical data that led to another ongoing trial (NCT02555644) investigated the efficacy of CHK1 inhibitors in combination with EGFR targeted therapies and/or radiotherapy (Zeng *et al*., 2017). In this study, prexasertib combined with EGFR targeting therapies significantly decreased cell proliferation and delayed tumor growth in both HPV‐positive and HPV‐negative head and neck squamous cell carcinoma mouse models (Zeng *et al*., 2017). These promising results provided rationale to test CHK1 inhibitors in combination with both genotoxic and nongenotoxic therapy regimens for cancer treatment.

## Concluding remarks

Standard clinical testing of CRC includes identifying mutations in oncogenes such as *KRAS* and *BRAF*, as well as characterization of the microsatellite status. Currently, the status of DNA repair genes is not investigated in CRCs. However, MSS CRCs carry a higher proportion of mutations in HR genes, and defects in this pathway have been associated with genomic instability. Whole‐genome sequencing analysis of breast cancer samples can identify tumors that exhibit genomic rearrangements due to functional deficiencies in homologous recombination even *BRCA* wild‐type cells, and these characteristics can be used to predict therapy response to PARP inhibitors. We propose that, in addition to genetic screening for mutations in known DNA repair genes, identification of gene alterations and genomic rearrangements indicative of a repair‐defective phenotype should be performed systematically in CRC patients. Characterizations based on functional repair deficiency, rather than analyses based primarily on genetic alterations, are likely to better predict therapy response to inhibitors of DNA repair pathways in CRC patient cohorts.

## Author contributions

NMR wrote the manuscript. LN performed bioinformatics analysis of The Cancer Genome Atlas datasets. FDN and AB contributed to the manuscript preparation.

## Conflicts of interest

The authors declare no conflict of interest.
